# Heterochronic microRNAs in temporal specification of neural stem cells: application toward rejuvenation

**DOI:** 10.1038/npjamd.2015.14

**Published:** 2016-01-07

**Authors:** Takuya Shimazaki, Hideyuki Okano

**Affiliations:** 1 Department of Physiology, Keio University School of Medicine, Tokyo, Japan

## Abstract

Plasticity is a critical factor enabling stem cells to contribute to the development and regeneration of tissues. In the mammalian central nervous system (CNS), neural stem cells (NSCs) that are defined by their capability for self-renewal and differentiation into neurons and glia, are present in the ventricular neuroaxis throughout life. However, the differentiation potential of NSCs changes in a spatiotemporally regulated manner and these cells progressively lose plasticity during development. One of the major alterations in this process is the switch from neurogenesis to gliogenesis. NSCs initiate neurogenesis immediately after neural tube closure and then turn to gliogenesis from midgestation, which requires an irreversible competence transition that enforces a progressive reduction of neuropotency. A growing body of evidence indicates that the neurogenesis-to-gliogenesis transition is governed by multiple layers of regulatory networks consisting of multiple factors, including epigenetic regulators, transcription factors, and non-coding RNA (ncRNA). In this review, we focus on critical roles of microRNAs (miRNAs), a class of small ncRNA that regulate gene expression at the post-transcriptional level, in the regulation of the switch from neurogenesis to gliogenesis in NSCs in the developing CNS. Unraveling the regulatory interactions of miRNAs and target genes will provide insights into the regulation of plasticity of NSCs, and the development of new strategies for the regeneration of damaged CNS.

## Introduction

The discovery of neural stem cells (NSCs) in the developing and adult brain of mammals, including human, has raised expectations for their clinical application in cell replacement therapies for central nervous system (CNS) disorders.^1–4^ Numerous studies have sought to elucidate mechanisms of self-renewal and differentiation of NSCs, as control of neural stem cell fate is required to obtain desired types of neurons and glia. However, the developmental reduction of plasticity and neuropotency of NSCs is a limiting factor in use of *in vitro* expanded NSCs and mobilization of latent and active NSCs in the adult CNS. The differentiation potential of NSCs is spatiotemporally regulated during CNS development, enabling these cells to generate various types of neurons and glia in appropriate regions and time points to enable the formation of complex neural networks.^5,6^ In the developing cerebral cortex, NSCs sequentially generate several different types of neurons specifically located to form the six distinct layers and finally differentiate into glia after ceasing neurogenesis.^3,5^ This is normally a one-way process. The developmental potential of NSCs is restricted in a time-dependent manner and becomes much more gliogenic both *in vivo* and *in vitro,*^5,6^ which may be a feature of the aging process in NSCs. One proposed solution to this problem is the use of embryonic stem cells (ESCs) and induced pluripotent stem cells (iPSCs)^7^ as sources for highly plastic NSCs in an early developmental state. Direct reprograming of non-neural somatic cells and other types of neural cells into specific types of neurons is also an option.^8,9^ However, safety concerns remain surrounding the use of these cells.^10,11^ In particular, the quality of neural cells derived via reprogramming has not been definitively confirmed. Elucidation of regulatory mechanisms underlying the temporal specification of NSCs to identify means of controlling NSC plasticity *in vivo* and *in vitro* is thus important to increase the viability of cell replacement therapy using NSCs. Under these circumstances, an increasing number of studies designed to elucidate mechanisms behind the temporal specification of NSCs have been conducted over the past decade.^5,6^ In particular, the mechanisms by which neurogenesis precedes gliogenesis during the CNS development have been intensively studied. In these studies, several transcription factors and non-coding RNAs (ncRNAs) including microRNAs (miRNAs), which comprise a class of small ncRNAs, typically 21–25 nucleotides in length that regulate gene expression at post-transcriptional level by binding to the 3′-untranslated region (UTR) of specific target messenger RNAs (mRNAs) have been identified as critical regulatory factors.

In mammals, the initial transcript containing miRNAs is transcribed mostly by RNA polymerase II from the exons and introns of coding genes, or from intergenic regions encoding long ncRNAs containing one or more miRNAs. A single strand of the mature miRNA duplex, is incorporated into the RNA-induced silencing complex (RISC) containing Dicer, a ribonuclease III enzyme, and Argonaute 2 protein (Ago2). The miRNA–RISC complex recognizes mRNA targets through partial base pairing to the seed sequence, which is 6–8 nucleotides mostly located at positions 2–8 from the 5′-end of miRNA, and mediates their repression through translational repression, mRNA degradation, or mRNA deadenylation. Each miRNA recognizes multiple target transcripts and each mRNA transcript is targeted by multiple miRNAs. Details on the biogenesis and functions of miRNA, including non-canonical miRNA, have been reviewed elsewhere.^12,13^

Hundreds of miRNAs are detected in the developing and adult mammalian CNS, and some of these have been shown to have critical roles in CNS development.^14–17^ Initially, a series of loss-of-function (LOF) analyses of Dicer, including its conditional deletion using various specific Cre drivers, revealed that miRNAs confer robustness to CNS development.^14^ For instance, the conditional deletion of Dicer in neural progenitors, including NSCs, using *Emx1*-Cre or *Nestin*-Cre drivers resulted in significant progenitor cell death in the developing cortex and defects in neurogenesis and gliogenesis.^18–20^ In recent years, studies of the specific functions of miRNAs in the CNS development have been increasing. Several recent studies revealed the presence of complex networks formed by heterochronic genes, including several transcription factors, miRNAs, and epigenetic regulation, in the process of the neurogenesis-to-gliogenesis transition by NSCs.

## Regulation of gliogenic competence by miR-153 and miR-17/106

**Figure 1:**
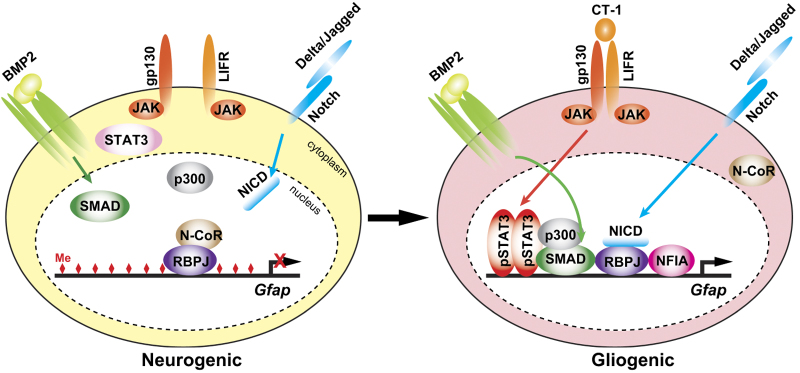
Temporal regulation of *Gfap* gene transcription in NSCs in response to gliogenic signals. In the early-neurogenic period, the *Gfap* promoter including STAT3-binding site in NSCs is highly methylated (diamonds in red), severely limiting access by transcriptional activators downstream of gliogenic signals. Moreover, repressor complex containing N-CoR, a co-repressor, may associate with RBPJ on the promoter.^70–72^ After acquisition of gliogenic competence, NFIA induces demethylation of the *Gfap* promoter, and the activation of JAK/STAT signaling via gp130/LIFR by increase of a ligand CT-1 secreted from neurons leads to the formation of STAT3/SMAD/p300 complex on the promoter in co-operation with BMP signaling. The translocation of N-CoR to the cytoplasm may allow association of NICD to RBPJ, resulting in derepression and activation of *Gfap* promoter in the astrocytic differentiation.

**Figure 2:**
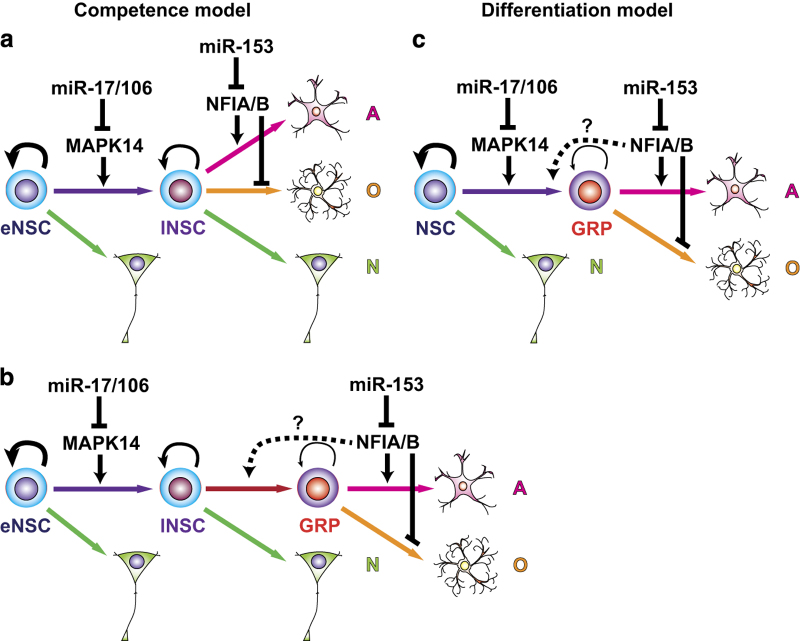
Schematic models for the acquisition of gliogenic competence and involvements of miR-17/106-MAPK14 axis and miR-153-NFIA/B axis. (**a,** **b**) Competence models: early-neurogenic NSCs (eNSC), which generate only neurons (N) become late-gliogenic NSCs (lNSC) acquired gliogenic competence to differentiate into astrocytes (A) or oligodendrocytes (O) directly and/or indirectly through the glial-restricted progenitor (GRP) state in response to gliogenic signals. miR-17/106b may be required for the maintenance of eNSC state via repression of MAPK14. miR-153 represses NFIA/B expressions which are essential for astroctytic differentiation of NSCs and/or GRPs. It is not clear whether NFIA/B are required for the differentiation of NSCs into GRPs. (**c**) Differentiation model: NSCs rarely differentiate into GRPs in the early-neurogenic period. miR-17/106b may be required for the maintenance of neuropotency and/or inhibition of differentiation of NSCs into GRP via repression of MAPK14. The role of miR-153-NFIA/B axis is same as that in the competence models. NFIA has also been shown to suppress oligodendrocyte differentiation.

The initiation of gliogenesis by NSCs proceeds in two steps: acquisition of gliogenic competence and induction of differentiation.^5,21^ In the developing mammalian CNS, NSCs acquire gliogenic competence at midgestation, and subsequently oligodendrogliogenesis begins.^22^ Astrocytic differentiation can be detected only in the perinatal period.^21^ The gliogenic competence is defined as the competence to respond to gliogenic signals that induce glial differentiation in NSCs. NSCs in the early-neurogenic phase before midgestation cannot differentiate into glia, even in the presence of these signals.^22–26^ The interleukin-6 (IL-6)/Janus kinase (JAK)/signal transducer and activator of transcription (STAT) pathway, BMP2/4/mothers against decapentaplegic homolog (SMAD) pathway, and the Notch signaling pathway are known as major inductive signals for astrocytic differentiation (Figure 1).^5,21^ IL-6 family cytokines such as ciliary neurotrophic factor), leukemia inhibitory factor, and cardiotrophin-1 (CT-1) activate the JAK family of non-receptor tyrosine kinases via binding to the receptor complex, which shares a common receptor subunit glycoprotein 130 (gp130), inducing activation of STAT1 and STAT3.^27^ In particular, developmental increase of CT-1 caused by increase of neurons that express CT-1 is critical for the timing of astrocytic differentiation.^28^ BMP2/4 bind to a tetrameric complex of type I and type II serine/threonine kinase receptors (BMPR1/2), thereby activating SMAD transcription factors.^29^ In canonical Notch signaling of vertebrates, binding of ligands such as Delta and Jagged to Notch receptors at the cell surface leads to nuclear translocation of Notch intercellular domain (NICD) after proteolytic cleavage. The NICD is subsequently translocated into the nucleus and forms a transcriptional complex with the coactivator Mastermind and the DNA-binding protein recombination signal binding protein for immunoglobulin kappa J region (RBPJ)/CBF1/Su(H)/Lag-1 (CSL) to activate the transcription of target genes, including glial fibrillary acidic protein (*Gfap*).^30^ The BMP/SMAD and JAK/STAT pathways have been shown to facilitate astrocytic differentiation of NSCs synergistically via the co-operative action of SMAD1 and STAT3 via complex formation with a transcriptional coactivator p300 to induce the expression of astrocyte-specific genes.^31,32^ In the early-neurogenic phase, before the acquisition of gliogenic competence, the activation of BMP/SMAD pathway and JAK/STAT pathway cannot induce differentiation of NSCs into astrocytes.^22–26^ BMPs rather facilitate neuronal differentiation of NSCs at this stage.^33^ The epigenetic modification of glia-specific genes is a part of the regulation of gliogenic competence. The promoter region of the *Gfap* gene including the STAT3-binding site is epigenetically silent; high levels of DNA methylation and repressive histone marks are found in NSCs during the early-neurogenic phase (Figure 1).^22,25,34^ Several transcription factors have been shown to be involved in this competence change in developing NSCs. At midgestation, the expression of a SRY (Sex determining Region Y)-box 9 (SOX9) a SOX (SRY-like HMG box) family transcription factor increases and induces an expression of the transcription factor nuclear factor I (NFI) A, which is involved in the initiation of gliogenesis.^35–37^ NFIA forms a complex with SOX9 to induce a subset of glial-specific genes including GFAP.^37^ NFIA has also shown to mediate Notch signaling-induced demethylation of *Gfap* gene promoter in NSCs.^38^ Recently, our group identified miR-153 a CNS specific evolutionally highly conserved miRNA as a modulator of the neurogenesis-to-gliogenesis switch by targeting NFIA and NFIB which has also been shown to be required for astrogliogenesis (Figure 2).^39–41^ In the developing mouse CNS, miR-153 is broadly expressed throughout the CNS, including the ventricular zone (VZ). However, the expression level of miR-153 in cortical neural stem and/or progenitor cells (NSPCs) markedly decreases at midgestation. Overexpression (OE) of miR-153 results in inhibition of astrogliogenesis and maintenance of NSPCs in an undifferentiated state in ESC-derived NSPC cultures and in the developing cortex. Conversely, inhibition of miR-153 in early-neurogenic NSPCs induces precocious expression of NFIA/B and astrogliogenesis. As the presence of NFIA/B is essential for gliogenic competence of NSPCs, miR-153 must be one of critical factors for the timing of astrogliogenesis via modulation of gliogenic competence. Intriguingly, miR-153 expression is maintained in the VZ of the lateral ganglionic eminence (LGE), even at the perinatal stage.^41^ As the LGE and, subsequently, the adult subventricular zone (aSVZ) constantly generate neurons migrating into olfactory bulbs throughout life,^42^ miR-153 may also be involved in the maintenance of neurogenic NSPCs. Indeed, miR-153 OE in ESC-derived NSPCs and cortical NSPCs resulted in enhanced neurogenesis to a limited extent along with the maintenance of an undifferentiated state of NSPCs.^41^ It could therefore be argued that the constant neurogenesis in the aSVZ is a form of neoteny, which may be supported by the expression of miR-153.

We also identified miR-17 and 106a/b (miR-17/106), belonging to the miR-17 family of miRNAs that share a common seed sequence as critical regulators for neurogenesis-to-gliogenesis switch in NSPCs downstream of chicken ovalbumin upstream promoter-transcription factor I and II (COUP-TFI and COUP-TFII, also known as NR2F1 and NR2F2), which belong to the orphan nuclear hormone receptor family (Figure 2).^43^ Ahead of this discovery, we had found that the transient increase of COUP-TFI/II expression in NSPCs at midgestation is essential for their temporal specification, including acquisition of gliogenic competence.^22^ The double knockdown (KD) of *Coup-tfI/II* in ESC-derived NSPCs and the developing mouse forebrain resulted in sustained neurogenesis and the prolonged generation of specific types of neurons, which are normally born only at the early neurogenic stage. Initially, miR-17/106 was found to be expressed in NSPCs specifically at the early-neurogenic stage and increased in response to the *Coup-tfI/II* KD in ESC-derived NSPCs. Our functional analyses revealed a time-dependent decrease of miR-17/106 expression in developing NSPCs, leading to an increase of one of their targets, mitogen-activated protein kinase 14 (MAPK14, also known as p38α) in turn, which is essential for the acquisition of gliogenic competence. Importantly, miR-17-mediated OE or LOF of MAPK14 in stage-progressed highly gliogenic NSPCs resulted in robust recovery of neuropotency, which is literally an instance of ‘rejuvenation’. However, miR-17 OE did not alter the temporal change in the epigenetic status of *Gfap* promoter.

These results suggest that the acquisition of gliogenic competence involves at least two distinct regulatory layers, regulation of the expression of glial-specific genes, including epigenetic regulation, which involves regulation by the Notch-NFIA and miR-153-NFIA/B axes, and regulation of NSPC neuropotency by the miR-17/106-MAPK14 axis. The former is currently only observed in the regulation of astrogliogenesis. In fact, miR-153 OE does not alter oligodendrogliogenesis in ESC-derived NSPCs (Tsuyama *et al.*, unpublished result). In the latter, miR-17/106 may be essential for the maintenance of neuropotency in NSPCs, as the neurogenic phenotype induced by miR-17 OE in developing and highly gliogenic NSPCs is extremely pronounced compared with that induced by miR-153 OE, and even resembles to that of early-neurogenic NSPCs (Figure 2a,b). Moreover, miR-17 has been shown to suppress astrocytic differentiation of mouse cortical progenitors via repression of BMPR2, which is acquired in cortical VZ after midgestation and is essential for BMP2/4 signaling, which inhibits neurogenesis and promotes astrogliogenesis.^44^ If that is the case, then MAPK14 may be required for the competence change of NSCs to allow glial differentiation in the presence of sufficient amounts of gliogenic factors, including NFIA/B and gliogenic signals. The miR-17 OE in ESC-derived NSPCs does not alter *Nfia/b* expression (Naka-Kaneda *et al.*, unpublished result). Alternatively or additionally, miR-17/106 may suppress differentiation of NSCs into glial lineages independent of the epigenetic regulation of glial-specific genes (Figure 2c). In this case, the expression level of MAPK14 may determine the frequency of the commitment into glial lineages and NFIA/B may simply be required for astrocytic differentiation of glial-restricted precursors (GRPs), because NFIA OE suppresses differentiation of oligodendrocyte progenitor cells into oligodendrocytes. It is possible that the majority of undifferentiated NSPCs increased as a result of miR-153 OE^41^ are GRPs and/or astrocyte precursors. To date, there is no reliable marker to distinguish these glial progenitor cells from NSCs, although GRPs can be isolated *in vitro.*^45,46^ We also do not know whether astrocyte and oligodendrocyte lineages can be differentiated directly from NSCs, or must necessarily pass through the GRP state. Future studies regarding glial development should thus include a focus on identification of definitive NSCs and/or GRP markers.

## Regulation of neuronal and glial differentiation by miRNAs

miRNAs are also involved in the terminal differentiation of neurons and glia at appropriate time points. Initial analyses of Dicer conditional knockout (CKO) mice using *Nestin*, *Emx1*-, *Foxg1*- and *Olig1*-Cre drivers revealed that the contribution of miRNAs to neuronal and glial differentiation in the developing CNS is more pronounced after mid-gestation.^14,18–20^ In particular, the massive reduction of gliogenesis in the developing spinal cord was observed in the CKO mice with *Nestin*-Cre or *Olig1*-Cre driver,^47,48^ whereas no major defect in neurogenesis was observed in any of these mice, although massive cell death and reduced maturation of neurons were evoked in the developing cortex of Dicer-CKO mice with *Nestin*, *Emx1*-, and *Foxg1*-Cre drivers.^14,18–20^ The relatively normal neurogenesis in these Dicer-CKO mice in early developmental stages may be owing to the presence of key miRNAs for both promotion of neurogenesis (e.g., miR-9 and miR124)^17^ and promotion of self-renewal and proliferation of NSPCs (e.g., miR-19 and miR-92)^49,50^ (see also other reviews for more details).^15,16,51^ In contrast, a mosaic deletion of Dicer in the developing cortex in mouse by the transfection of a Cre-expressing plasmid resulted in prolonged neurogenesis, but no significant alteration in gliogenesis.^52^ The phenotypic discrepancy between the mosaic deletion and CKO using several drivers indicates that miRNAs are involved in both the cell autonomous and non-cell autonomous regulation of NSPC differentiation.

**Figure 3:**
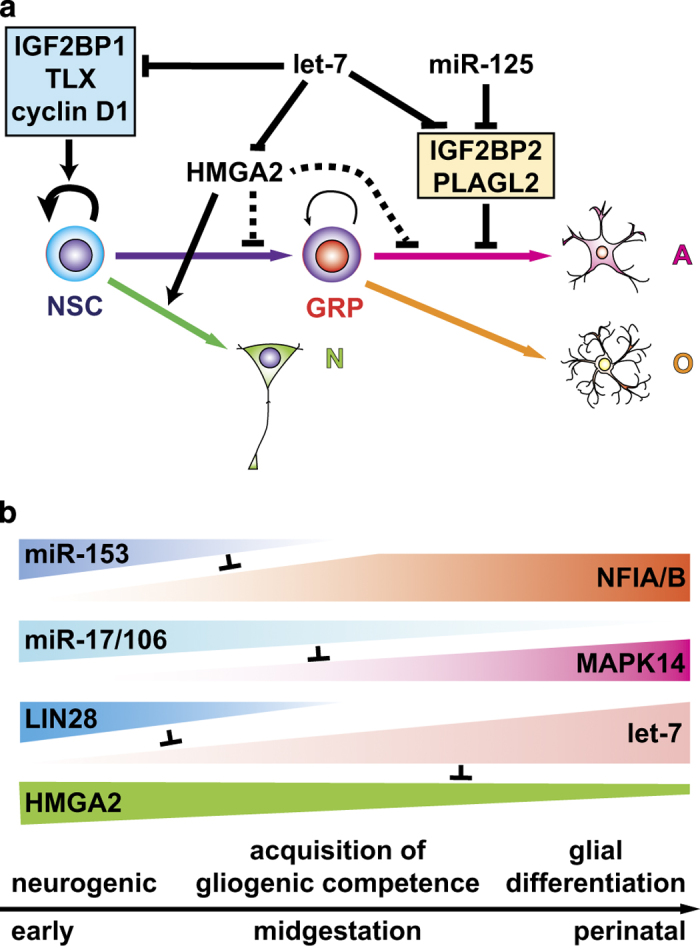
Roles for miRNAs in the regulation of neurogenesis-to-gliogenesis switch by NSCs. (**a**) Roles of *let-*7 and miR-125 in the neuronal and glial differentiation of NSCs. *let-*7 suppresses self-renewal and neuronal differentiation of NSCs via repression of IGF2BP1, TLX, cyclin D1, and HMGA2, respectively, but facilitate astrocytic differentiation of GRPs via repression of IGFBP2 and PLAGL2, which also are targets of miR-125. (**b**) A schematic model for the regulation of neurogenic-to-gliogenic switch by heterochronic genes including microRNAs in the developing NSCs. The expressions of miR-153 and miR-17/106 decline with time, leading to increases of their target NFIA/B and MAPK14, respectively, which are essential for gliogenesis. The *let-*7 expression increases with time caused by decrease of LIN28, leading to a inhibition of neurogenesis via repression of HMGA2. A, astrocyte; N, neuron; NSCs, neural stem cells; O, oligodendrocyte.

Among several dozens of specific miRNAs involved in neurogenesis and gliogenesis, the *let-*7 family of miRNAs seems to have a key role in the timing of glial differentiation (Figure 3a). *let-*7 miRNAs are heterochronic genes encoding components of machinery underlying timing mechanisms in animal development, and were first identified as genes that control the timing of cell-fate decisions in the larval development of *Caenorhabditis elegans.*^53^ In mammals, *let-*7 miRNAs have been shown to be involved in a wide range of biological processes, including differentiation and temporal specification of several types of stem cells.^54^ In NSC development, *let-*7b was initially found to be involved in the age-dependent decline of stem cell function by targeting high-mobility group-AT-hook 2 (HMGA2) a member of the high-mobility group A (HMGA) family, which encodes a small non-histone chromatin-associated protein that modulates transcription of many genes by altering chromatin structure.^55^ Age-dependent increase of mature *let-*7 miRNAs in NSPCs after midgestation seems to contribute to the age-dependent decline of HMGA2, which induces upregulation of the cell cycle inhibitors p16^INK4a^ and p19^ARF^. *let-*7b has also been shown to induce differentiation of NSPCs into neurons and glia by targeting an orphan nuclear receptor TLX and cyclin D1 (Figure 3a).^56^ Moreover, deletion of Insulin-like growth factor two mRNA binding protein 1 (IGF2BP1; also known as IMP-1, ZBP1), another target of *let-*7 miRNAs in mice, reduces proliferation of NSPCs and induces premature differentiation of neurons and astrocytes along with a reduction of HMGA2 expression in the developing cortex, while *let-*7g OE exhibits a similar phenotype with a significant reduction of IGF2BP1 expression.^57^ Gain-of-function (GOF) and LOF phenotypes of HMGA2, however, do not completely fit in the expected phenotypes as a target of *let-*7 miRNAs. In the mouse embryonic cortex and human NSPC cultures derived from hPSCs, HMGA2 appears to suppress astrocyte differentiation but be required for neurogenesis.^58,59^ This is unsurprising, as *let-*7 miRNAs regulate multiple target genes, as described above. Overexpression of *let-*7 miRNAs suppresses neuronal differentiation, depending on the cellular context. In the neurogenesis from hESC-derived NSPCs and zebrafish retinal injury, *let-*7 miRNAs are likely to target a neurogenic basic helix-loop-helix transcription factor Achaete-scute homolog 1 to inhibit neurogenesis^60^ or prevent premature Müller glia dedifferentiation upon retinal injury.^61^ Intriguingly, Cimadamore *et al.*^60^ reported that *let*-7 activity was not detected in hESC-derived NSPCs owing to the high expression level of LIN28, a well-known inhibitor of *let-*7 biogenesis driven by SOX2. This finding suggests that these NSPCs were in an early-neurogenic phase because the temporal patterns of LIN28 and mature *let-*7 miRNA expression in developing NSPCs are inversely correlated. SOX2 and LIN28 are downregulated along the neuronal differentiation of hESC-derived NSPCs, thereby *let-7* is derepressed in differentiating neurons. Phenotypes induced by the ectopic expression of particular genes are often inconsistent with their physiological functions. Recently, *let-*7 and miR-125 have been shown to promote astrocytic differentiation of GRPs derived from mouse ESCs through the regulation of multiple targets in parallel with JAK/STAT pathway (Figure 3a).^62^ Moreover, *let-*7 OE in hPSC-derived NSPCs enhanced oligodendrogliogenesis.^56^ Thus, *let-*7 miRNAs are involved in gliogenesis at multiple levels. The developmental increase of *let-*7 miRNAs in NSPCs thus seems to be integrated into the timing mechanisms of gliogenesis (Figure 3b).

## Conclusion

A recent large body of work on miRNAs has revealed their significant contribution to a wide range of biological processes, including cell-fate determination events. One might also argue that the major function for miRNAs may be buffering stochastic noise in the gene expression,^63^ as LOF of individual miRNAs often results in mild or no phenotype under normal physiological conditions.^64^ This may be due in part to compensation by other miRNAs targeting the same mRNAs.^65^ In contrast, GOF of a miRNA causing a significant reduction of the expression of multiple target often results in a clear phenotypic change.^66^ However, as described above, several miRNAs have critical roles in the regulation of neurogenesis-to-gliogenesis switch in the developing CNS (Figure 3b). Similarly, *let-*7, miR-125, and miR-9 have also been found to have major roles in the temporal specification of retinal progenitors.^67^ Inhibition of all these or *let-*7 activity leads to the prolonged generation of ganglion cells from progenitors stuck in an early state. Moreover, evidence is accumulating for the involvement of specific miRNAs in the age-related changes in the properties of several tissue stem cells such as hematopoietic stem cells and mesenchymal stem cells in addition to NSCs.^54,68^ Aging usually causes reduction of regenerative capacity of stem cells.^69^

The limited regenerative capacity of adult tissues in mammals compared with that of other lower vertebrates, such as urodele amphibians and teleost fish, may largely be due to the limited number and plasticity of tissue stem cells. Therefore, rejuvenation of adult stem cells represents an important target in regenerative medicine and aging research. Given the robust contribution of miRNAs to the temporal specification of several tissue stem cells including NSCs, controlling stem cell plasticity via LOF and GOF of specific miRNAs along with the mobilization of stem cells may serve as a possible therapeutic approach to various degenerative diseases. Further studies to elucidate how miRNAs regulate plasticity of stem cells during aging will provide important information for better understanding of stem cell aging and development other therapeutic approaches including use of small molecules that modulate functions and/or expressions of specific targets of key miRNAs.
